# INSIDE-OUTSIDE COMMUNITY ADVISORY BOARDS: ONE MODEL FOR ENGAGING OLDER BIPOC AND WOMEN IN CLINICAL RESEARCH

**DOI:** 10.1093/geroni/igac059.1680

**Published:** 2022-12-20

**Authors:** Jennifer James

**Affiliations:** University of California, San Francisco, San Francisco, California, United States

## Abstract

Historical legacies of unethical research performed on people in prison, coupled with stringent policies intended to protect those who are incarcerated from exploitation, has meant that much clinical research about the health of people who are incarcerated is conducted at a distance, without directly engaging those who are incarcerated in the formulation of studies. As a result, research conducted about correctional healthcare and the healthcare needs of incarcerated persons may not reflect specific stakeholder values or priorities. In this session, we will describe barriers in engaging incarnated older women and BIPOC patients, their healthcare providers, and their loved ones in the research process and new models for the development of research questions that center stakeholder perspectives. We will describe identified priority areas for future research, insight on the ethics of research consent and participation with this population, and methodological considerations for clinical, social, and behavioral research with this vulnerable population.

